# A Student’s Viewpoint on ChatGPT Use and Automation Bias in Medical Education

**DOI:** 10.2196/57696

**Published:** 2024-04-15

**Authors:** Jeanne Maria Dsouza

**Affiliations:** 1 Kasturba Medical College, Manipal, India

**Keywords:** AI, artificial intelligence, ChatGPT, medical education

The editorial *ChatGPT in Medical Education: A Precursor for Automation Bias?* by Nguyen [1] is very timely, appropriate, and informative. Being a medical student myself, I find that it gives a balanced view on the use of ChatGPT, which is sweeping across the globe at a spectacular pace. One of the hallmarks of this tool is that it is almost universally accessible, even in parts of the world where there may be limited access to quality medical education. As authors have rightly pointed out, ChatGPT is useful for summarizing information, generating practice questions, and giving instantaneous feedback [2-4], and it could serve as an effective personalized tutor. It provides high-quality scientific text gleaned from a quick and comprehensive review of the literature and presents text in an efficient, readable, and versatile style [1]. It is no wonder that it is gaining immense popularity among students, including medical students, who are “burdened with the impossible task of balancing the need to continuously learn and retain competencies and the need to provide compassionate patient care,” as aptly underscored in the editorial [1].

The downside of this powerful tool has also been well portrayed. There is a very real risk of automation bias, especially among medical students in the younger generation, who are digitally savvy but often lack experience and confidence in their clinical skills. The blind dependence on ChatGPT and other artificial intelligence (AI) tools could corrode their thinking and decision-making skills and lead to erroneous medical outcomes. The clinical setting is undoubtedly the best classroom for students to develop the skills for understanding and accommodating the needs, expectations, and values of patients and their caregivers in the real-world scenario, as well as cultivate leadership qualities and work in a team. It is vital for us students to retain our originality, identity, and critical analytical skills to avoid falling into the trap of AI solutionism.

The need for AI education at this crucial juncture has been well brought out. At present, only a minority of students have received AI education [5]. Incorporating it into the medical curriculum is a challenging, multidisciplinary endeavor. Knowing how and when to use this powerful tool in a responsible manner, without clouding clinical judgment and in keeping with the tenets of medical ethics, is paramount. I agree with Nguyen’s [1] view that ChatGPT should be used as a supplementary tool rather than as the default resource for medical education. There is a need to exercise vigilance in the utilization of this tool right from the formative years of medical professionals.

AI is here to stay, and ChatGPT will undoubtedly have an all-pervading influence on medical education and the practice of medicine itself. Therefore, its optimal utilization is the need of the hour. Imparting AI education would help unleash the power of ChatGPT, but appropriate pre-emptive measures to keep its disruptive potential in check are needed to pave the way for an AI-savvy generation of medical professionals with sound clinical judgment and skills.

## Abbreviations

AI: artificial intelligence
